# Familial Adenomatous Polyposis-Associated Desmoids Display Significantly More Genetic Changes than Sporadic Desmoids

**DOI:** 10.1371/journal.pone.0024354

**Published:** 2011-09-09

**Authors:** Els Robanus-Maandag, Cathy Bosch, Saeid Amini-Nik, Jeroen Knijnenburg, Karoly Szuhai, Pascale Cervera, Raymond Poon, Diana Eccles, Paolo Radice, Marco Giovannini, Benjamin A. Alman, Sabine Tejpar, Peter Devilee, Riccardo Fodde

**Affiliations:** 1 Department of Human Genetics, Leiden University Medical Center, Leiden, The Netherlands; 2 Center for Human Genetics, University of Leuven, Leuven, Belgium; 3 Department of Molecular Cellular Biology, Leiden University Medical Center, Leiden, The Netherlands; 4 INSERM U674, Fondation Jean Dausset-CEPH, Paris, France; 5 Division of Orthopaedic Surgery and Department of Surgery, University of Toronto and the Hospital for Sick Children, Toronto, Canada; 6 Wessex Clinical Genetics Service, Southampton University Hospitals Trust, Southampton, United Kingdom; 7 Department of Preventive and Predictive Medicine, Fondazione IRCCS Istituto Nazionale dei Tumori, Milan, Italy; 8 Department of Pathology, Josephine Nefkens Institute, Erasmus University Medical Center, Rotterdam, The Netherlands; Ohio State University Medical Center, United States of America

## Abstract

Desmoid tumours (also called deep or aggressive fibromatoses) are potentially life-threatening fibromatous lesions. Hereditary desmoid tumours arise in individuals affected by either familial adenomatous polyposis (FAP) or hereditary desmoid disease (HDD) carrying germline mutations in *APC*. Most sporadic desmoids carry somatic mutations in *CTNNB1*. Previous studies identified losses on 5q and 6q, and gains on 8q and 20q as recurrent genetic changes in desmoids. However, virtually all genetic changes were derived from sporadic tumours. To investigate the somatic alterations in FAP-associated desmoids and to compare them with changes occurring in sporadic tumours, we analysed 17 FAP-associated and 38 sporadic desmoids by array comparative genomic hybridisation and multiple ligation-dependent probe amplification. Overall, the desmoids displayed only a limited number of genetic changes, occurring in 44% of cases. Recurrent gains at 8q (7%) and 20q (5%) were almost exclusively found in sporadic tumours. Recurrent losses were observed for a 700 kb region at 5q22.2, comprising the *APC* gene (11%), a 2 Mb region at 6p21.2-p21.1 (15%), and a relatively large region at 6q15-q23.3 (20%). The FAP-associated desmoids displayed a significantly higher frequency of copy number abnormalities (59%) than the sporadic tumours (37%). As predicted by the *APC* germline mutations among these patients, a high percentage (29%) of FAP-associated desmoids showed loss of the *APC* region at 5q22.2, which was infrequently (3%) seen among sporadic tumours. Our data suggest that loss of region 6q15-q16.2 is an important event in FAP-associated as well as sporadic desmoids, most likely of relevance for desmoid tumour progression.

## Introduction

Desmoid tumours are locally aggressive but histologically benign fibrous tumours also known as deep fibromatoses or aggressive fibromatoses [1]. Despite this benign classification, both local infiltration and risk of recurrence following excision lead to a significant increase in morbidity and mortality. Desmoids are relatively rare tumours accounting for only 0.03% of all neoplasms. They arise in connective tissues and are thought to be of mesenchymal origin [2]. Prevalent sites are the abdominal region, shoulder girdle, chest wall, and inguinal regions [3].

Desmoids occur either as part of a hereditary predisposition or in sporadic patients. Familial adenomatous polyposis (FAP) is an autosomal dominant disorder predisposing to the development of multiple colorectal adenomatous polyps during adolescence. FAP affects on average 1 in 8,000 individuals and is characterised by extra-colonic manifestations. One of these, desmoid disease, occurs in about 12% of FAP kindreds, leading to a 1000-fold increased risk of desmoid development in FAP patients compared to the general population. FAP desmoids often occur in the abdomen after prophylactic colectomy [4]. This post-operative occurrence, together with the local infiltration and high risk of recurrence makes desmoid disease the most common cause of death among FAP patients [1].

FAP-associated desmoid tumours are caused by germline mutations in the tumour suppressor gene *APC*, located on chromosome 5q22.2, followed by somatic inactivation of the wild-type *APC* allele by mutation or deletion [5]–[8]. The main APC's tumour suppressing function resides in its capacity to regulate the Wnt signal transduction pathway [9]. In the absence of Wnt signals, a dedicated complex of proteins, including APC, axin and glycogen synthase kinase-3β (GSK-3β) phophorylates β-catenin, resulting in its ubiquitylation and degradation by the proteasome. Signalling by Wnt factors inhibits the APC complex. As a result, β-catenin is stabilised and translocates into the nucleus, where it interacts with nuclear TCF/LEF transcription factors. These TCF/LEFs inhibit specific target genes when bound to Groucho/TLE repressors, while association with β-catenin blocks these interactions and converts TCF/LEFs into transcriptional activators to drive the transcription of the target genes. In FAP patients, mutational activation of the Wnt signalling pathway due to *APC* inactivation in intestinal epithelial cells results in inappropriate activation of TCF4 and initiation of adenoma formation. We have previously reported on a novel syndrome, hereditary desmoid disease (HDD) where multifocal desmoid tumours are inherited as an autosomal dominant trait with 100% penetrance in a three-generation kindred [10]. HDD segregated with an unusual 3′ *APC* mutation at codon 1924, confirming that mutations in this specific part of the gene are associated with desmoid tumour formation [11]. Whereas a minority of sporadic desmoids have somatic *APC* mutations, most of them carry oncogenic *β-catenin* gene *(CTNNB1)* mutations [12]. Instead of TCF4, TCF3 is frequently activated in these tumours [13]. Thus, dysregulation of Wnt signalling is likely to underlie desmoid formation in the vast majority of both hereditary and sporadic cases.

Cytogenetic analysis, including interphase fluorescence *in situ* hybridisation (FISH), and chromosome comparative genomic hybridisation (chromosome CGH) have been employed to determine the spectrum of genomic alterations of a limited number of mainly sporadic desmoid tumours. Common gains have been described for chromosomal regions 1q21 [14] and 9p12 [14], and whole chromosomes 8 [15]–[17] and 20 [14]–[18]. Common losses have been reported for regions on chromosome arms 5q [14], [16], [17] and 6q [14], [17], [18]. However, both genome-wide screening techniques have disadvantages: cytogenetic analysis is based on short cultures of tumour cells with the risk of introducing additional *de novo* alterations, whereas chromosome CGH has limited resolution (≥10 Mb) to detect copy number abnormalities (CNAs). Recently, Salas et al. used array CGH to analyse the genomic alterations in a large series of desmoid tumours [19]. They confirmed loss of 6q, loss of 5q, gain of 20q, and gain of chromosome 8 as recurrent alterations in these tumours. However, in their series, only 3 of 10 FAP-associated desmoids displayed genomic alterations.

In this study, we report on a genome-wide screen using high-resolution array CGH (∼1 Mb) of CNAs in a series of desmoid tumours with a relatively high contribution of FAP-associated cases. We compared the frequencies of genomic alterations among FAP-associated and non-FAP-associated (sporadic) cases. In addition, by combining our data with those of others [14]–[19], we could further demarcate the chromosomal segments important in desmoid tumourigenesis.

## Materials and Methods

### Tumour Samples

Fifty-three fresh frozen tumour samples were collected at four institutes: Center for Human Genetics, University of Leuven, Leuven, Belgium (21 samples); INSERM U674, Fondation Jean Dausset-CEPH, Paris, France (15 samples); Hospital for Sick Children, Toronto, Canada (13 samples); and the Italian Registry of Hereditary Colorectal Cancer, Milan, Italy (Dr. L. Bertario, 4 samples). In addition, DNA of two fresh frozen tumours, HDD-H of patient III:2 and HDD-I of patient III:6, of our hereditary desmoids disease (HDD) family was available (Table 1 and S2) [10]. In this family, multifocal desmoid tumours were inherited as an autosomal dominant trait, and HDD segregated with a 3′ *APC* mutation at codon 1924. For the latter reason, they were classified as FAP tumours in this study. Three FAP tumours were derived from a family with 2 relatives in the study (D15-1 and D15-2 of a male patient and D16 of his sister). Colonic polyposis had been observed in 12 FAP patients, not in the 2 HDD-FAP patients and not in patient D15. The germline *APC* mutation was known in 14 of 17 FAP-associated tumours. In 28 of 38 non-FAP-associated desmoid tumours, the *β-catenin* gene *(CTNNB1)* mutation in exon 3 was known and in 1 of 38 tumours an *APC* mutation was present (data not shown). The remaining tumours were characterised as FAP or non-FAP based on clinical data and positivity of tumour cells upon immunostaining for β-catenin. DNA was extracted from the tumour samples according to standard methods.

**Table 1:** Copy number abnormalities detected by array CGH in individual desmoid tumors.

Category	Case	Sex	Mutation	Location	Copy number abnormalities
Non-FAP	P9A	F	*CTNNB1* na, *APC* na	Intra-abd small bowel	−6p21.2-p12, −6q12-qter
	P15A	F	*CTNNB1*	Abd wall	−6q12-qter, +21q22.11-q22.12
	P18A	F	*CTNNB1*	Abd wall recurrence	−6, −13
	D17	M	*APC*	Abd wall post-surgery	−5q22.2-q23.1, −6p21.2-p21.1
	D8	F	*CTNNB1*	Extra-abd paravertebral	−5q14.3-q22.1, −5q35.1, +8
	D12	F	*CTNNB1*	Extra-abd paraspinal	+20
	D6	F	no *CTNNB1*, *APC* na	Extra-abd rectus abdominus	+13q12.12-q12.13
	183T	F	no *CTNNB1*, no *APC*	Extra-abd back	−6
	141T	M	*CTNNB1*	Extra-abd arm	−6
	D11	M	*CTNNB1*	Extra-abd foot	+12q14.3-q15
	D7	M	*CTNNB1*	Extra-abd leg	+8, +20
	114T	M	*CTNNB1*	Extra-abd leg	+8q
	D5	M	*CTNNB1*	Extra-abd suprapubical	−6
	129T	M	no *CTNNB1*, no *APC*	Extra-abd back	+8q, +Yq11.223-q11.23
FAP	HDD-H	F	*APC*	Intra-abd	−5q21.1-q31.1
	D15-1	M	*APC*	Intra-abd	−5q11.2-qter, −6q14.1-q24.3, −8p21.3-p11.21, −Yq11.223-q11.23
	D15-2	M	*APC*	Intra-abd	+1p13.3-p13.2, −6, −Y
	2444M	M	*APC*	Intra-abd	−10q22.3, +X
	P13A	F	*APC*	Abd wall	−6q12-qter, −13q14.11-q34, +20q
	P6A	F	*APC*	Abd wall	−5q22.1-q22.2, −13
	P5A	F	*APC*	Extra-abd inguinal canal	−5q15-q31.2
	2428M	M	*APC*	Abd wall	−6
	P8A	M	*APC*	Abd wall	−3p26.1-p23, −6q15-q23.3, +20cen
	D16	F	*APC*	Extra-abd shoulder/back	−5q14.3-q33.1, −X

Abbreviations: F, female; M, male; na, not available; Intra-abd, intra-abdominal; Abd, abdominal; Extra-abd, extra-abdominal.

### Ethics Statement

The research was performed at the Department of Human Genetics of the Leiden University Medical Center (LUMC), Leiden, The Netherlands. Clinical samples were irreversibly anonymised and results of scientific research could not be linked to individual patients. The Committee Medical Ethics of the Leiden University Medical Center specifically waived approval for this study because it falls under paragraph 7∶467 Civil Law Code of The Netherlands.

### Array CGH and Image Analysis

Arrays and the array CGH procedure have been described previously [20]. In brief, the array comprised spotted DNA from ∼3,500 large insert genomic clones at an average spacing of about 1 Mb throughout the genome (Wellcome Trust Sanger Institute set, information on this clone set at http://www.ensembl.org/Homo_sapiens) and was produced at the Leiden University Medical Center, as described previously [20]. Approximately 400 ng DNA was used per labeling. Cy3-labeled test (tumour) and Cy5-labeled reference (normal) DNAs were hybridised to the arrays together with herring sperm and Cot-1 DNA. Arrays were scanned with a DNA microarray scanner (Agilent Technologies, Amstelveen, The Netherlands) or GenePix Personal 4100A scanner (Axon Instruments, Westburg BV, Leusden, The Netherlands). Spot intensities were measured using GenePix Pro 4.1 software (Axon Instuments). Within this software, spots in which the reference DNA intensity was either below five times the average of the background or presented more than 3% saturated pixels were excluded from further analyses. The test/reference ratios were normalised for the median of the ratios of all features. The triplicates of the features were averaged in a homemade routine developed in Microsoft Excel 2000, and spots outside the 20% confidence interval of the average of the replicates were excluded from further analyses. Data points were included when at least 2 spots passed the criteria. CNAs, i.e. gains or losses, were determined based on the combined use of the array CGH-Smooth tool [21] and visual inspection. In the latter method, regions of CNA were assessed by subjective judgment in which all clones in a particular region were noticeably above or below the baseline of the log2 ratios of that region in other tumours. CNAs on chromosomes 5 and 6 were validated by MLPA analysis, see below. Excluded from the array CGH analysis were subtelomeric and pericentromeric regions, with highly repetitive sequences, and genomic variable regions (http://www.tcag.ca). The array CGH data are described in accordance with MIAME guidelines and have been deposited in the NCBI Gene Expression Omnibus (GEO) database under accession no. GSE28458.

### MLPA

The multiplex ligation-dependent probe amplification (MLPA) procedure was performed as described, using 100 ng tumour DNA [22], [23]. Probes within each set were designed to produce PCR products ranging in size from 86 to 122 bp and with a minimum separation of 2 bp (Table S1). The hybridising regions of the probes had a Tm of at least 69°C, with a GC content between 35 and 60%, and lacked 4 or more successive identical nucleotides. For each product, the presence of duplicated and/or repetitive sequences was excluded using the BLAT program (http://genome.ucsc.edu). The downstream oligonucleotide of each pair was 5′ phosphorylated to allow ligation to occur. Two probe set mixes were used, one mix including *FER* without *AMD1*, and the other including *AMD1* without *FER*. All reagents for the MLPA reactions were purchased from MRC-Holland (Amsterdam, The Netherlands). Data were analysed with GeneScan® Analysis Software (Applied Biosystems, Foster City, CA) and exported to Microsoft Excel.

Raw data of each sample were normalised as described [24]. In short, for each sample, all peak heights of the focus probes were divided by the average peak height of the two reference probes, resulting in normalised peak heights for each focus probe. For the 16 control samples (desmoid tumours without detectable CNA(s) as determined by array CGH) the median normalised peak height for each probe was taken, being equivalent to the presence of two copies for each probe. For the desmoid tumour samples with detectable CNA(s), a ratio was calculated by dividing the normalised peak height of a focus probe in the tumour with the median normalised peak height of that probe in the control samples. Loss of one copy of a focus probe was defined as ratio <0.8.

### Statistics

The presence of differences in tumour location between FAP and non-FAP patient groups was tested for by chi-square analysis. Logistic regression was used to examine the effect of tumour location (intra-abdominal, abdominal wall and extra-abdominal) on the presence of CNAs in FAP-associated desmoid tumours. Results were considered significant at *p*<0.05.

## Results

### Clinical Characterisation of Patients

A total of 55 desmoid tumours obtained from 21 males (including one male with two tumours) and 33 females were incorporated in this study (Table 2). The previously reported predominance of desmoids among women versus males was confirmed in our study population (60% versus 40%) [3]. Of the FAP cases, 8 (47%) were male and 9 (53%) female; of the non-FAP cases 14 (37%) were male and 24 (63%) female. Thus, the female/male ratio for FAP cases was 1.13 compared to the female/male ratio of 1.71 for non-FAP cases, indicating a more equal distribution of desmoids among males and females with FAP.

**Table 2:** Characteristics of patients with desmoid tumor (n = 55).

Category	Subcategory	FAP (%)	Non-FAP (%)	Total (%)
Location	Intra-abdominal	10 (59)	5 (13)	15 (27)
	Abdominal wall	5 (29)	10 (26)	15 (27)
	Extra-abdominal	2 (12)	23 (61)	25 (46)
M/F	Male	8 (47)	14 (37)	22 (40)
	Female	9 (53)	24 (63)	33 (60)
Mean age at diagnosis (range)		23.6 (0–44)	32.7 (2–67)	

Approximately 59% (10/17) of the desmoids diagnosed in FAP patients were in the abdomen versus just 13% (5/38) in non-FAP patients (Table 2). In contrast, extra-abdominal desmoids were diagnosed in 61% (23/38) of non-FAP patients and just 12% (2/17) of FAP patients. In both patient groups, the frequency of desmoids in the abdominal wall was similar (29% (5/17) in FAP patients and 26% (10/38) in non-FAP patients). Overall, desmoids in the abdominal region (intra-abdominal and in the abdominal wall) comprised nearly all FAP desmoids and extra-abdominal desmoids comprised the majority of non-FAP desmoids, a significant difference in our study population (*p* = 0.001).

The average ages of desmoid tumour diagnosis were similar for FAP and non-FAP patients (23.6, range 0–44, and 32.7, range 2–67, respectively) (Table 2).

### Array CGH Analyses

The 55 desmoid tumours were analysed by array CGH. Thirty-one of these desmoids (56%) displayed a normal copy number profile (Table S2). A limited number of CNAs was found in the remaining 24 desmoids: on average 1.8 CNAs per tumour (Table 1).

In particular non-FAP profiles were normal (24 of 38 tumours, 63%), whereas the majority of FAP profiles showed CNAs (10 of 17 tumours, 59%). The difference in CNA frequency was statistically significant after correction for anatomical location: the odds for CNAs was almost 7 times as high for FAP-associated tumours compared to sporadic cases (OR = 6.97, 95% CI 1.23–39.56, *p* = 0.028).

**Figure 1:**
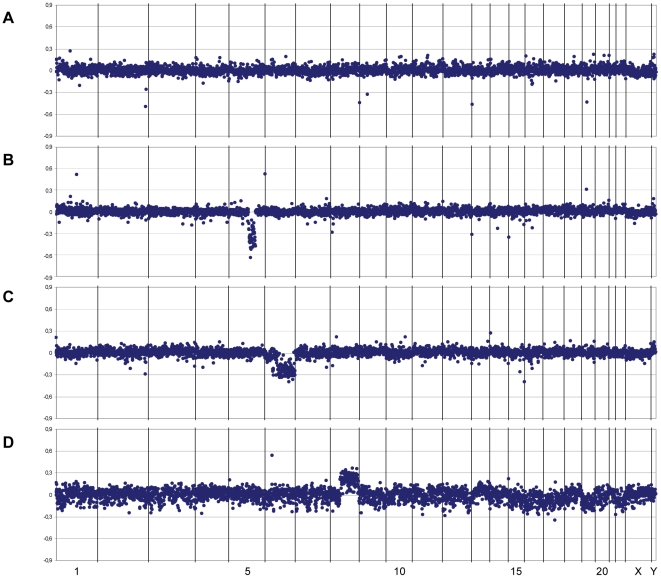
Examples of copy number abnormalities identified by array CGH analysis. Graphs show the log_2_ ratio profiles for the BACs aligned along the X-axis from chromosome 1 to chromosome Y. (A) FAP-associated desmoid tumour HDD-I without any CNAs. (B) FAP-associated desmoid tumour HDD-H with loss of the 5q21.1-q31.1 region involving the *APC* gene. (C) Non-FAP-associated desmoid tumour P9A with loss at 6p21.2-p12 and 6q12-qter. (D) Non-FAP-associated desmoid tumour 114T with gain of chromosome 8q.

Examples of individual array CGH profiles are given in Figure 1. The array CGH profiles of both desmoid tumours from two FAP-associated HDD patients differed markedly: no CNAs could be detected in the profile of tumour HDD-I (Figure 1A), whereas the profile of tumour HDD-H showed solely loss of 5q21.1-q31.1 (Figure 1B). Figure 1C demonstrates losses of 6q12-qter and, less obvious, 6p21.2-p12 in tumour P9A of a non-FAP patient. The latter loss could be confirmed by MLPA analysis (see below). In the tumour obtained from case 114T only gain of the long arm of chromosome 8 could be detected (Figure 1D).

**Figure 2:**
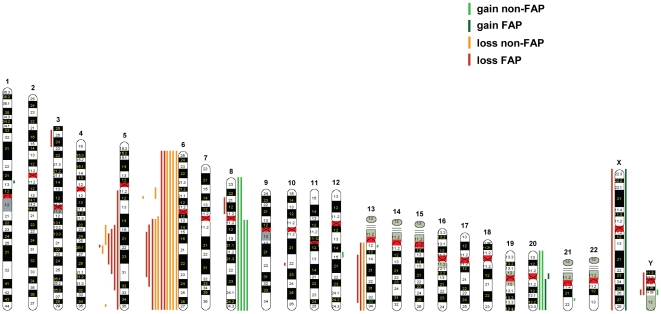
Composite array CGH profiles. The results for all chromosomes of the genome are presented with red lines indicating loss and green lines indicating gain. Losses and gains are shown for non-FAP-associated desmoid tumours (light color) and for FAP-associated desmoid tumours (dark color).

Recurrent gains (≥3 cases) were restricted to chromosomes 8 and 20 (Figure 2, Table 3). Whole chromosome gains were noted in 2 cases for chromosome 8 and in 2 cases for chromosome 20, including one case with gain of chromosomes 8 plus 20. In addition, gain of chromosome arm 8q was observed in 2 cases and gain of 20q in 1 case. Altogether, we found gain of 8q in 4/55 (7%) and gain of 20q in 3/55 cases (5%). Interestingly, 5 of these 6 samples with gain of 8q and/or 20q were sporadic desmoid tumours with extra-abdominal locations, thereby representing a frequent event in this group (5 of 23; 22%).

**Table 3:** Minimal recurrent regions of gain and loss detected by array CGH in desmoid tumors.

Chromosomal region of gain (+) or loss (−)	Flanking clones	Position (Mb)	FAP (%) n = 17	Non-FAP (%) n = 38	Total (%) n = 55
+8q	RP11-567J20 - RP11-370K2	47.8–146.2	0 (0)	4 (11)	4 (7)
+20q	RP11-348I14 - RP13-152O15	21.8–62.4	1 (6)	2 (5)	3 (5)
−5q22.2	RP11-3B10 - RP11-467F22	112.0–112.7	5 (29)	1 (3)	6 (11)
−6p21.2 – p21.1	RP3-350J21 - RP11-162O6	39.0–41.0	2 (12)	6 (16)	8 (15)
−6q15 – q23.3	RP1-122O8 - RP11-323N12	90.4–135.7	5 (29)	6 (16)	11 (20)
−13q14.11 – q34	RP11-117I13 - RP11-40E6	42.2–109.4	2 (12)	1 (3)	3 (5)

Recurrent losses (≥3 cases) only involved chromosomes 5, 6, and 13 (Figure 2, Table 3). Among FAP-associated desmoids, the highest frequency of loss was seen for regions on chromosomes 5 and 6, each in 5 of 17 cases (29%). The smallest region of overlap for the losses on chromosome 5 was a 700 kb segment at 5q22.2, harbouring the *APC* gene. In addition, two sporadic desmoid tumours (D8 and D17) showed deletion of distinct 5q regions (Table 1, Figure 2). Deletion of the 5q region in tumour D17, together with a somatic *APC* mutation, resulted in loss of *APC*. However, the deletion in tumour D8, previously found to carry a *β-catenin* gene mutation, was positioned at 5q14.3-q22.1, and encompass BACs directly centromeric to the *APC* gene but not those comprising the 5′ and 3′ end of the *APC* gene (RP11-3B10 and CTC-1554D6). Chromosome 6 was found to be involved at the highest overall frequency, occurring in 11 out of 55 (20%) cases (Figure 2, Table 3). The main recurrent region of loss could be derived from the array CGH data to extend from 6q15 to 6q23.3. Deletion of this relatively large region of 45 Mb was more frequent among FAP-associated tumours (5/17, 29%) compared to sporadic cases (6/38, 16%). The sole region deleted on chromosome 6 of non-FAP tumour D17 was a 2 Mb segment in 6p21.2-p21.1. A second deleted region in 6p21.2-p12 in non-FAP tumour P9A overlaps this segment (Table 1, Figure 2). These findings may indicate the presence of a second region of recurrent loss on chromosome arm 6p involved in 8 of 55 (15%) desmoids. Finally, chromosome 13 showed a low frequency of losses (3 of 55 cases, 3%), with 13q14.11-q34 as the minimal recurrent deleted segment (Figure 2, Table 3).

### MLPA

**Figure 3:**
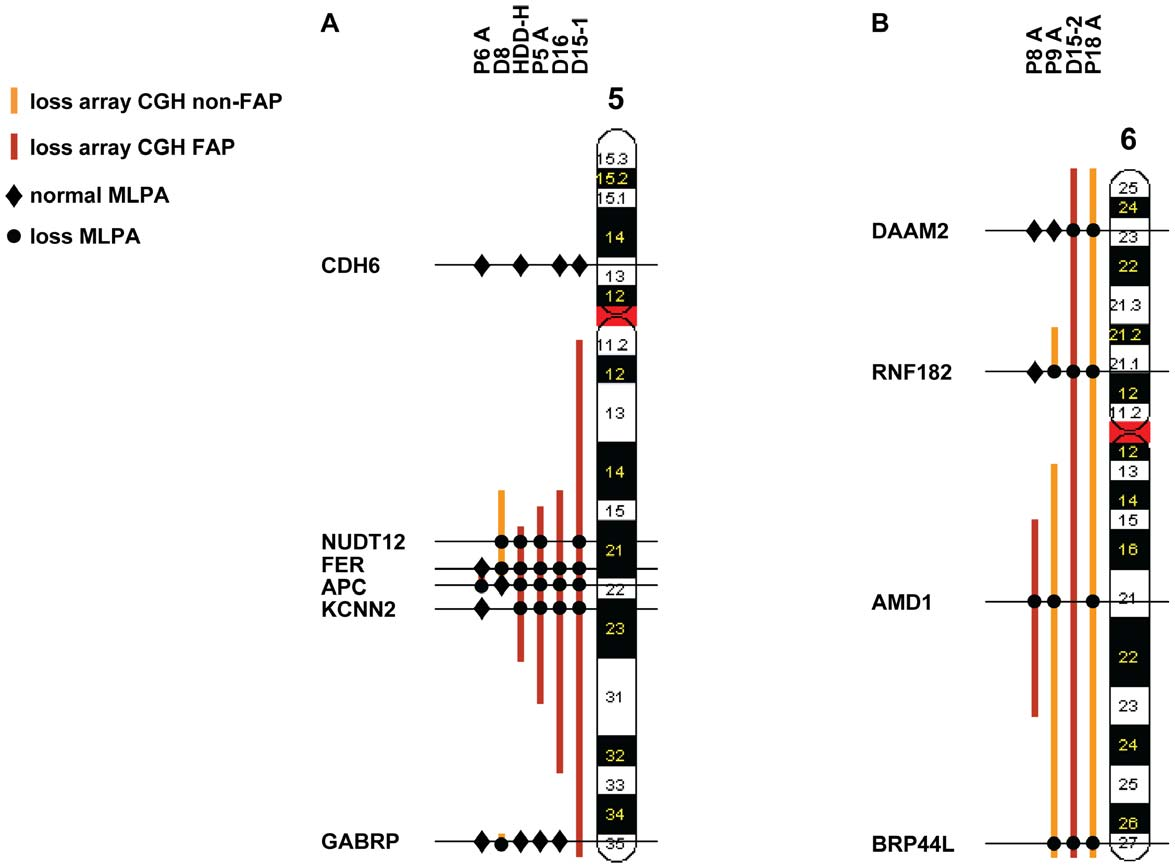
MLPA data overview verifying losses on chromosomes 5 and 6 as detected by array CGH analysis. The MLPA results for the indicated gene probes are presented with dots and diamonds, indicating loss and no loss, respectively. Losses on chromosomes 5 (A) and 6 (B) as detected by array CGH are shown for non-FAP-associated desmoid tumours (light red color) and FAP-associated desmoid tumours (dark red color).

To verify the extent of the losses on chromosomes 5 and 6, MLPA probes were designed and used to assess the copy number status of critical regions on these chromosomes. The positions of these probes and their copy number status in the respective tumours are indicated in Figure 3. In general, the array CGH-assessed losses on chromosomes 5 and 6 in the tumours could be confirmed by the MLPA analyses. In particular, inclusion of the *APC* gene in the small 5q22.1-q22.2 segment deleted in tumour P6A and its exclusion from the deleted 5q14.3-q22.1 segment in tumour D8 could be confirmed. Moreover, the additional loss of the small 5q35.1 segment in the latter tumour was substantiated by the MLPA analysis with the GABRP probe (Figure 3A). Unfortunately, due to lack of DNA, the small 6p21.2-p21.1 segment lost in tumour D17 could not be analysed by MLPA. However, loss of the overlapping 6p21.2-p12 segment in tumour P9A was substantiated by MLPA analysis with the RNF182 probe (Figure 3B).

## Discussion

The desmoid tumours here investigated by array CGH frequently lack genomic alterations (56%), while those tumours with CNAs harbour only a limited number of changes (less than two per tumour). These results are remarkable, since desmoids display aggressive and infiltrative growth and local recurrence causing considerable morbidity and mortality. The sporadic tumours displayed a significantly lower frequency of CNAs (37%) than the FAP-associated desmoids (59%). Hence, our data confirm the general lack of genomic abnormalities in sporadic desmoid tumours previously reported by others [14], [18], [19], with the novel addition of the high frequency of CNAs in FAP-associated desmoid tumours. Analysis of a total of 40 desmoid tumours using low-resolution chromosome CGH revealed that 4 of 5 FAP- and 12 of 23 sporadic desmoids lacked CNAs [14], as well as 10 of 12 non-described desmoids in an additional study by the same group [18]. The recent study by Salas et al., using array CGH on 194 desmoid tumours, revealed 76% of the tumours to lack chromosomal changes (7 of 10 FAP-associated and 141 of 184 non-FAP-associated tumours) [19]. Our finding of a high frequency of CNAs in FAP-related desmoids versus a low frequency in non-FAP-related tumours was significant after correction for anatomical location. This correction was based on the unbalanced contribution of extra-abdominal tumours which were overrepresented in the sporadic desmoid group and underrepresented among the FAP-associated desmoids, as is the case in general. The increased occurrence of CNAs in FAP-associated tumours with mutations in the *APC* gene *versus* the reduced occurrence in sporadic tumours harbouring mainly *β-catenin* gene mutations is in agreement with the notion that loss of the mitotic function of APC may lead to chromosomal instability [25].

Based on their infrequent detection of CNAs in abdominal desmoids, Brandal et al. hypothesised that these are, in contrast to extra-abdominal tumours with more frequent CNAs, non-neoplastic tissues or tumours with genetic changes too small to be discovered by chromosome CGH [18]. However, using the more sensitive array CGH method, we found no significant differences in the frequency of occurrence of CNAs between the various locations of the tumours: CNAs were detected in 12 of 25 extra-abdominal tumours and in 12 of 30 abdominal tumours.

By array CGH, we further demarcated previously described CNAs and identified novel chromosomal aberrations. The limited number of recurrent CNAs found in our study was restricted to regions on chromosomes 5, 6, 8, 13 and 20. These regions frequently encompassed whole chromosomes or chromosome arms, as exemplified by the two recurrent gains that we observed, namely of chromosome 8(q) and of chromosome 20(q). Involvement of chromosome 8 has long been an apparent discrepancy in genetic studies of desmoids. Cytogenetic and FISH analyses have repeatedly revealed trisomy 8 [15]–[17], [26], whereas chromosome CGH studies indicated a normal number of chromosome 8 [14], [18]. The more sensitive array CGH analysis indisputably reveals gain of chromosome 8 (or its q arm) in our study (11%) and, although at a lower frequency (3%), also in that of Salas et al. [19]. Gain of chromosome 20(q), in our study in 5% of cases, has been found in previous studies at varying frequencies (7–56%) [14]–[19], [26]. Remarkably, nearly all gains of chromosomes 8(q) and 20(q) in our study proved to be present in sporadic desmoids with an extra-abdominal location. Gains of chromosome arms 8q and 20q have also been reported for gastric and colorectal cancers [27]–[29]. In desmoid tumours, trisomies 8(q) and 20(q) may be disease modulating secondary events in addition to primary molecular genetic aberrations, as has been described for trisomy 8 in acute myeloid leukemia (AML) [30].

Using chromosome CGH and DNA extracted from formalin-fixed paraffin-embedded (FFPE) tumour tissue, Larramendy et al. found frequent gains at 1q21 (39%) and 9p12 (21%) in the 28 desmoids they examined [14]. In contrast to this, we and also Salas et al. [19], studying in total almost 250 frozen tumour samples, did not detect these genomic alterations. Therefore, it is likely that the reported gains at 1q21 and 9p12 were attributable to undefined changes introduced into the DNA during the fixation process.

Our array CGH and MLPA data demonstrate the frequent involvement (29% of cases) of chromosome region 5q22.2 in the losses on the long arm of chromosome 5 in FAP-associated desmoid tumours (Table 3, Figure 3A). The smallest region of recurrent loss, defined by flanking clones RP11-3B10 and RP11-467F22 in tumour P6A, has a length of 700 kb and harbours only a few genes, including *APC*. To our knowledge, this is the smallest segment shown to be frequently deleted on chromosome arm 5q in desmoid tumours [14]–[19], further supporting the notion that *APC* is the main target gene in this segment. Thus, in the FAP-associated desmoid tumours from individuals carrying a germline *APC* mutation, complete *APC* inactivation through loss of the second, wild-type allele was a relatively frequent event (29%). Among the sporadic tumours, loss at 5q22.2 occurred only in the single case with a known somatic *APC* mutation (tumour D17), but not in the vast majority of cases carrying a *β-catenin* gene mutation. The observed mutual exclusivity of *β-catenin* and *APC* gene mutations is consistent with their equivalent effects on β-catenin stability and Tcf transactivation (Table 1 and S2) [31]. This is furthermore illustrated by the sporadic desmoid tumour D8 carrying a *β-catenin* gene mutation. This tumour harboured a deletion centromeric to, but not including *APC* as demonstrated by the array CGH and MLPA data depicted in Figure 3A. Part of this deletion was shared by 4 other FAP-associated desmoid tumours including the FAP-associated HDD-H tumour. The recurrently deleted region at 5q21.1-q22.1 (∼14 Mb) may encompass the desmoid modifier gene that we previously proposed to be linked to *APC* in our hereditary desmoid disease (HDD) family [10]. The *FER* gene at 5q21.3 in this region encodes Fes-related protein, which is known to interact with β-catenin [32]. In *Caenorhabditis elegans*, the ortholog of human FER represses Wnt signalling in collaboration with the ortholog of human APC, making *FER* an attractive candidate modifier gene in this region [33].

The second region with a high frequency of loss in FAP-associated desmoids (29%) at 6q15-q23.3 (45 Mb) was also, though less frequently, lost in the non-FAP-associated tumours in our panel (16%). In previous studies, Larramendy et al. and Salas et al. defined minimal recurrent region of loss at 6q15-q21 and 6q14-16.2, respectively [14], [19]. Combining the latter and our data, we conclude that loss of the smallest region of overlap at 6q15-q16.2 is another critical event in desmoid tumourigenesis. This 12.5 Mb region includes several genes with known or potential tumour suppressor function: *ANKRD6*, *BACH2*, and *MAP3K7/TAK1* at 6q15, and *EPHA7* and *NLBP*/*KIAA0776* at 6q16.1. The protein product of *ANKRD6* is directly involved in Wnt signalling: ANKRD6 suppresses Wnt signals mediated by the β-catenin pathway via recruitment of Casein kinase Iα to the β-catenin degradation complex [34]. Loss of heterozygosity for *BACH2*, a transcription repressor gene, has been detected in non-Hodgkin's lymphomas [35]. Deletion of a small region at 6q15 including the *MAP3K7*/*TAK1* gene, which encodes the TAK1 protein, involved in transforming growth factor-β-induced signalling pathways, has been associated with high-grade prostate cancers [36]. *EPHA7* encodes an ephrin/Eph receptor, which is a positive regulator of apoptosis during forebrain neurogenesis and could potentially be involved in oncogenesis [37]. The protein product of the *NLBP*/*KIAA0776* gene has been recently identified as a novel LZAP-binding protein. Based on lack of expression in hepatocellular carcinoma cells with strong invasive activity it has been suggested that NLBP may act as a novel tumour suppressor by inhibiting cell invasion, blocking NF-κB signalling, and increasing stability of the LZAP protein [38]. Loss of 6q16.1 is also a frequent finding in osteosarcomas [39]. Moreover, deletion at 6q15-16.1 has been identified in childhood T-cell acute lymphoblastic leukemia (T-ALL) as a predictor of poor early treatment response [40].

Another potential region of recurrent loss, at 6p21.2-p21.1, was found at considerable frequency (15%) in the FAP- as well as non-FAP-associated desmoids in our series. Although this small segment (2 Mb), defined by the array CGH-derived deletion in chromosome arm 6p of tumour D17, could not be confirmed by MLPA because of lack of DNA, its loss was confirmed by MLPA in tumour P9A as part of a larger deletion (Figure 3B). This small region includes no known or potential tumour suppressor genes. The previous CGH studies did not reveal the sole loss of this small region on 6p, but reported loss of the entire chromosome 6 or loss of segments at more telomeric positions on 6p [14], [18], [19].

The infrequent loss (5% of cases) of most of chromosome arm 13q that we found was also identified in the study by Larramendy et al. [14], but was not observed in the work of Salas et al. [19].

In conclusion, the desmoid tumours displayed only a limited number of genomic alterations, including losses on chromosome arms 5q and 6q, and gains of chromosome arms 8q and 20q. In our panel, most FAP-associated tumours harboured a documented germline *APC* mutation, whereas most non-FAP associated tumours carried a *β-catenin* gene mutation, confirming that these genetic changes are primary events in desmoid tumourigenesis. The FAP-associated desmoids in our series displayed more frequent CNAs than the non-FAP-associated (sporadic) desmoids, which is in line with the notion that *APC* inactivation may lead to chromosomal instability [25]. In accordance with the tumour suppressor gene model, a high percentage of the FAP-associated desmoids showed loss of region 5q22.2, containing the *APC* gene, which was very infrequently seen in the non-FAP associated tumours. Our data and those of others suggest that loss of region 6q15-q16.1 is another important event in FAP- as well as non-FAP-associated desmoids [14], [19]. Frequent loss of this region has also been reported to occur in other types of cancer, indicating that this region contains a gene whose inactivation might be of relevance for the development of desmoids and other tumours as well.

## Supporting Information

Table S1 **MLPA probes.** F. probes, focus probes; R. probes, reference probes. For each probe, the first half-probe comprises the upstream hybridising sequence (**bold**) in addition to universal PCR primer A, the second half-probe comprises the downstream hybridising sequence (**bold**) in addition to universal PCR primer B.
(DOC)

Table S2 **Individual desmoid tumors without array CGH-derived copy number abnormalities.** Abbreviations: F, female; M, male; na, not available; Intra-abd, intra-abdominal; Abd, abdominal; Extra-abd, extra-abdominal.
(DOC)
