# Supplementary material for: Familial Adenomatous Polyposis-Associated Desmoids Display Significantly More Genetic Changes than Sporadic Desmoids
Source: PLoS One. 2011 Sep 9;6(9):e24354. doi: 10.1371/journal.pone.0024354 (PMC3170296; doi:10.1371/journal.pone.0024354)
Supplement: Table S1 — MLPA probes. F. probes, focus probes; R. probes, reference probes. For each probe, the first half-probe comprises the upstream hybridising sequence (bold) in addition to universal PCR primer A, the second half-probe comprises the downstream hybridising sequence (bold) in addition to universal PCR primer B. (DOC) [file pone.0024354.s001.doc]

**Table S1.** MLPA probes.

| **Category** | **Gene** | **Location** | | **PCR (bp)** | **Half-probe sequence** |
| --- | --- | --- | --- | --- | --- |
|  |  | **Cyto** | **Mb** |  |  |
| F. probes | *CDH6* | 5p13.3 | 31.2 | 96 | GGGTTCCCTAAGGGTTGGA**GATTATCTGGGACCACCACCGTGAA** |
|  |  |  |  |  | **CATCACACTGACTGATGTCAACGACAACC**TCTAGATTGGATCTTGCTGGCAC |
|  | *NUDT12* | 5q21.2 | 102.8 | 98 | GGGTTCCCTAAGGGTTGGA**GGAGAGAAGTAGAAGAGGAAAGTGGAGT** |
|  |  |  |  |  | **CAAAGTTGGCCATGTTCAGTATGTTGCT**TCTAGATTGGATCTTGCTGGCAC |
|  | *FER* | 5q21.3 | 108.3 | 122 | GGGTTCCCTAAGGGTTGGA**GTATATTTGATGAATACAGCCAGATAACCAGTCTTGTCA** |
|  |  |  |  |  | **CAGAGGAAATAGTGAATGTCCATAAAGAGATTCAAATGTCG**TCTAGATTGGATCTTGCTGGCAC |
|  | *APC* | 5q22.2 | 112.1 | 108 | GGGTTCCCTAAGGGTTGGA**TCCCTCCGTTCTTATGGAAGCCGGGAAGGATCT** |
|  |  |  |  |  | **GTATCAAGCCGTTCTGGAGAGTGCAGTCCTGTT**TCTAGATTGGATCTTGCTGGCAC |
|  | *KCNN2* | 5q22.3 | 113.8 | 114 | GGGTTCCCTAAGGGTTGGA**GCTGTATTCCTTAGCTCTGAAATGCCTTAT** |
|  |  |  |  |  | **CAGTCTCTCCACGATCATCCTGCTCGGTCTGATCATCGTGTA**TCTAGATTGGATCTTGCTGGCAC |
|  | *GABRP* | 5q35.1 | 170.2 | 104 | GGGTTCCCTAAGGGTTGGA**CCTTGCTAGAATATGCAGTTGCTCACTACAGTT** |
|  |  |  |  |  | **CCTTACAGCAGATGGCAGCCAAAGATAGG**TCTAGATTGGATCTTGCTGGCAC |
|  | *RNF182* | 6p23 | 13.9 | 106 | GGGTTCCCTAAGGGTTGGA**TGCTCTACTTCAGCTCCTTACCCTTAGGAAT** |
|  |  |  |  |  | **CTACTTACTGGTGTCTAAGAAAGTCACCCTTGG**TCTAGATTGGATCTTGCTGGCAC |
|  | *DAAM2* | 6p21.2 | 39.8 | 88 | GGGTTCCCTAAGGGTTGGA**CACAAGAAGGTGCTGCAGGCCAT** |
|  |  |  |  |  | **GCTGCACTACCAGGTGTATGCAG**TCTAGATTGGATCTTGCTGGCAC |
|  | *AMD1* | 6q21 | 111.2 | 122 | GGGTTCCCTAAGGGTTGGA**GGAAATTCTGATGAGTGAGCTTGACCCAGCAGTTATGGA** |
|  |  |  |  |  | **CCAGTTCTACATGAAAGATGGTGTTACTGCAAAGGATGTCA**TCTAGATTGGATCTTGCTGGCAC |
|  | *BRP44L* | 6q27 | 166.7 | 94 | GGGTTCCCTAAGGGTTGGA**CCACGCAACAAATGAAGTAGCCCAGCT** |
|  |  |  |  |  | **CATCCAGGGAGGGCGGCTTATCAAA**TCTAGATTGGATCTTGCTGGCAC |
| R. probes | *EXT2* | 11p11.2 | 44.2 | 86 | GGGTTCCCTAAGGGTTGGA**GTGTCCTGAGTGCACAGCCATA** |
|  |  |  |  |  | **GATGGGCTTTCACTAGACCAAA**TCTAGATTGGATCTTGCTGGCAC |
|  | *EP300* | 22q13.2 | 41.5 | 118 | GGGTTCCCTAAGGGTTGGA**CCAACCTAAGCACTGTTAGTCAGATTGATCCCAGCTCCAT** |
|  |  |  |  |  | **AGAAAGAGCCTATGCAGCTCTTGGACTACCCTATCA**TCTAGATTGGATCTTGCTGGCAC |

F. probes, focus probes; R. probes, reference probes. For each probe, the first half-probe comprises the upstream hybridizing sequence (**bold**) in addition to universal PCR primer A, the second half-probe comprises the downstream hybridizing sequence (**bold**) in addition to universal PCR primer B.
