# Supplementary material for: Familial Adenomatous Polyposis-Associated Desmoids Display Significantly More Genetic Changes than Sporadic Desmoids
Source: PLoS One. 2011 Sep 9;6(9):e24354. doi: 10.1371/journal.pone.0024354 (PMC3170296; doi:10.1371/journal.pone.0024354)
Supplement: Table S2 — Individual desmoid tumors without array CGH-derived copy number abnormalities. Abbreviations: F, female; M, male; na, not available; Intra-abd, intra-abdominal; Abd, abdominal; Extra-abd, extra-abdominal. (DOC) [file pone.0024354.s002.doc]

**Table S2.** Desmoid tumors without array CGH-derived copy number abnormalities.

| **Category** | **Case** | **Sex** | **Mutation** | **Location** |
| --- | --- | --- | --- | --- |
| Non-FAP | D10 | F | *CTNNB1* | Intra-abd spleen |
|  | 124T | F | *CTNNB1* | Intra-abd |
|  | D14 | F | *CTNNB1* | Intra-abd mesocolon |
|  | 58T | F | no *CTNNB1*, no *APC* | Intra-abd |
|  | D19 | F | *CTNNB1* | Abd wall anterior |
|  | D18 | F | *CTNNB1* | Abd wall anterior |
|  | P17A | F | *CTNNB1* | Abd wall |
|  | D20 | F | *CTNNB1* | Abd wall anterior |
|  | P12A | F | *CTNNB1* | Abd wall |
|  | P16A | F | no *CTNNB1*, *APC* na | Abd wall |
|  | P11A | F | no *CTNNB1*, *APC* na | Abd wall |
|  | D9 | F | *CTNNB1* | Extra-abd mandible |
|  | 181T | F | *CTNNB1* | Extra-abd thigh |
|  | 103T | F | *CTNNB1* | Extra-abd shoulder |
|  | 4T | F | *CTNNB1* | Extra-abd arm |
|  | D13 | F | *CTNNB1* | Extra-abd neck |
|  | D2 | F | *CTNNB1* | Extra-abd rectus abdominus |
|  | 65T | M | *CTNNB1* | Extra-abd thigh |
|  | 40T | M | *CTNNB1* | Extra-abd foot |
|  | 117T | M | *CTNNB1* | Extra-abd knee |
|  | P3A | M | *CTNNB1* | Extra-abd thoracic wall |
|  | P10A | M | *CTNNB1* | Extra-abd neck |
|  | D3 | M | no *CTNNB1*, *APC* na | Extra-abd leg |
|  | 180T | M | no *CTNNB1*, no *APC* | Extra-abd thigh |
| FAP | HDD-I | F | *APC* | Intra-abd |
|  | P2B | F | *APC* | Intra-abd small bowel |
|  | D1 | F | *APC* | Intra-abd |
|  | D4 | F | *APC* | Intra-abd |
|  | 2322M | M | *APC* | Intra-abd recurrence |
|  | FAP63M | M | *APC* | Intra-abd |
|  | P1A | M | *APC* | Abd wall |

Abbreviations: F, female; M, male; na, not available; Intra-abd, intra-abdominal; Abd, abdominal; Extra-abd, extra-abdominal.
